# One week versus three to five weeks of plaster cast immobilization for nonreduced distal radius fractures, a cost effectiveness analysis embedded in a stepped wedge cluster randomized controlled trial

**DOI:** 10.1007/s10198-025-01795-2

**Published:** 2025-06-02

**Authors:** Marcel A. N. de Bruijn, Emily Z. Boersma, Lysanne van Silfhout, Tjarda N. Tromp, Eddy M. M. Adang, Erik van de Krol, Michael J. R. Edwards, Vincent M. A. Stirler, Erik Hermans

**Affiliations:** 1 Department of Trauma Surgery, Radboud University Medical Center, Nijmegen, The Netherlands; 2 Department of IQ Healthcare, Radboud University Medical Center Nijmegen, Nijmegen, The Netherlands

**Keywords:** Distal radius fractures, Trauma, Conservative treatment, Cast immobilization, Cost effectiveness analysis

## Abstract

**Objective:**

Distal radius fractures are commonly seen at the Emergency Department. In the Netherlands, non- or minimally displaced fractures are immobilized for 3–5 weeks. However, evidence suggests shorter immobilization yields similar or better functional outcome. There is a lack of cost-effectiveness studies investigating shorter duration of cast immobilization. This study investigates the cost-effectiveness of one week of plaster cast immobilization versus three to five weeks.

**Methods:**

Cost-effectiveness data was collected as part of the Cast-OFF 2 study which started the 1st of January 2022. A randomized stepped wedge cluster design was used with 11 hospitals, and 10 clusters, including patients with an isolated non- or minimally displaced distal radius fracture without fracture reduction. Costs on medical consumption, and productivity were scored with the local Electronical Patient Record, and questionnaires at week one, six, month six, and twelve. Cost-effectiveness was reported per Quality-Adjusted Life Year (QALY).

**Results:**

A total of 402 patients were included (control *n = *197 vs intervention *n = *205). No differences in QALY were observed (+ 0.02, CI [-0.02, 0.06]). Cost savings for the intervention group ranged from €31.94 to €322.41 depending on different scenarios. The future perspective scenario with reduction of one outpatient clinic visit showed a significant cost saving of €254.27 (CI [-467.33, -41.21]). No significant differences were observed in baseline characteristics.

**Conclusion:**

One week of plaster cast immobilization for non- or minimally displaced distal radius fractures results in comparable or better cost savings compared to usual care. Adopting one week of cast immobilization as the new standard-of-care could reduce healthcare costs.

**Trial registration:**

Netherlands Trial Register NL9278.

CMO: 2–21-7308.

## Introduction

Distal radius fractures (DRF) represent a significant burden on the Emergency Department (ED), with approximately one-third of these fractures being non- or minimally displaced and without the need for fracture reduction [1]. The economic health-care costs of DRFs are substantial, with estimated health-care costs of 410 to 740 million United States dollar annually [2, 3].

As per standard-of-care in the Netherlands, these fractures are immobilized three to five weeks [4]. Several studies show that shorter immobilization is safe and results in the same or better functional outcome [5–9]. Patients may prefer shorter immobilization as they can resume daily activities sooner, if this does not lead to a worse functional outcome or more pain as mentioned in the Dutch DRF guidelines [4]. Furthermore, early removal of plaster cast immobilization is preferable by both patients and treating physicians, as longer immobilization can lead to muscle atrophy, persistent fracture pain, and pain at joint movement [10–12]. Potential financial advantages of shorter immobilization may be the result of less plaster change renewals, less hospital visits, and faster reintegration into daily activities thus resuming work activities sooner.

To date, there are no studies investigating the cost-effectiveness analysis (CEA) for patients treated with one week of plaster cast immobilization for the non- or minimally displaced DRF. Most literature is primarily focused on the surgical treatment and associating costs, or surgical in comparison to nonsurgical treatment [13]. Given the potential financial implications and patients preferences for shorter immobilization, there is a need to investigate the cost-effectiveness for these fractures. Shorter plaster cast immobilization may result in the same or better function compared to the standard-of-care, although its impact on healthcare is unclear. Shorter duration of immobilization with one week, could reduce the number of outpatient clinic visits during follow-up. This study will analyze the cost- effectiveness for nonoperative treatment of non- or minimally displaced DRF treated with one week of plaster cast immobilization compared to three to five weeks of plaster cast immobilization.

## Methods

### Setting and population

This cost-effectiveness analysis was conducted as part of the Cast-OFF 2 study, a stepped wedge cluster randomized controlled trial study comparing one week of plaster cast immobilization with three to five weeks for non-reduced distal radius fractures. The Cast-OFF 2 study recruited patients from January 2022 to January 2023 with a 12 month follow-up period, including 11 hospitals in the Netherlands (Appendix A). This study demonstrated the same if not better wrist function at six weeks following one week of plaster cast immobilization, with no differences in secondary dislocation or the need for surgical intervention compared to the control group [14]. A stepped wedge cluster randomized controlled design is an unidirectional cross-over design with hospitals transitioning from the standard three to five weeks immobilization to the intervention with one week of immobilization each month. Due to lower anticipated patient inclusions, alternations were made to the original stepped wedge design to optimize participant enrollment (Table 1).

**Table 1:** Cast-OFF 2 stepped wedge design, adjusted design. Protocol A: control group, three to five weeks of cast immobilization. Protocol B: intervention group, one week of cast immobilization. Cluster 1–10 represents 11 hospitals

Cluster	T1	T2	T3	T4	T5	T6	T7	T8	T9	T10	T11	T12	T13
1	A	A	B	B	B	B	B	B	B	B	B	B	B
2	A	A	A	A	A	A	A	A	A	B	B	B	B
3	A	A	A	A	A	A	B	B	B	B	B	B	B
4	A	A	A	A	B	B	B	B	B	B	B	B	B
5	A	A	A	A	A	A	B	B	B	B	B	B	B
6	A	A	A	A	A	A	B	B	B	B	B	B	B
7	A	A	A	A	A	A	A	B	B	B	B	B	B
8	A	A	A	A	A	A	A	A	B	B	B	B	B
9	A	A	A	A	A	A	A	A	A	B	B	B	B
10	A	A	A	A	A	A	A	A	A	A	B	B	B

Abbreviations: *T* Time in months

Inclusion criteria were patients with a non- or minimal displaced DRF, without fracture reduction or indication for surgical correction. Eligible patients were aged between 18 and 85 years old with an isolated non- or minimally displaced DRF, presenting at the ED within 72 hours post-injury, living independently and having an adequate understanding of the Dutch language.

Data for this CEA, including both direct medical costs and indirect healthcare related expenses, were collected next to the Cast-OFF 2 stepped wedge cluster randomized controlled trial.

### Economic evaluation

This CEA analyzed the costs, and cost-utility of three to five weeks compared with one week of plaster cast immobilization in patients with an isolated non- or minimally displaced DRF.

The EuroQol-5 Dimension-5 Level (EQ-5D-5L) health index, with the Dutch value set, was used to measure the quality of life, assessing five dimensions: mobility, self-care, usual activities, pain/discomfort, anxiety/depression, and with a patient’s self-rated health score on a visual analogue scale [15, 16]. Quality-adjusted-life-year (QALY) were calculated according to the trapezium rule. One QALY represents one year of perfect health.

### Resource utilization and unit costing

Costs were specified as direct costs, and indirect costs. Direct costs, or healthcare costs, included plaster cast treatment, ED visits, outpatient clinic follow-ups, diagnostic imaging, additional treatment for complications, hospital admissions, and the costs for healthcare professionals such as general practitioners, practice nurses, physiotherapists, company doctors, social workers, and the use of medication. Patient-specific costs were collected through cost-effectiveness questionnaires sent at week one, six, month six and twelve. Costs per unit were determined based on the most recent Dutch guideline for healthcare costing and medication pricing [17–21]. When available, mean costs for hospital treatment costs were used for both academical and peripheral hospitals. Medical consumption was calculated with an adjusted Medical Consumption Questionnaire [22]. Direct costs were calculated with the Dutch National Health Care Institute’s costing manual (2016/2024), and indexed for 2022 [23]. Due to unavailability of Silver Cross Prices for 2022, prices from 2023 were utilized [24].

Indirect costs, or societal costs, were estimated based on the loss or reduction in productivity resulting from fracture-related absence from work. Absence from work was calculated with questionnaires at week one, six, month six, and twelve using an adjusted Productivity Cost Questionnaire [22]. Sick leave and work absence were multiplied by the average cost per lost working hour [21]. Costs associated with pre-existing absence from work prior to the injury were not factored in the sum of total costs. Unit costing is shown in Table 2.

**Table 2:** Unit costing. Costs are indexed to 2022. Silver Cross Prices were only available for 2023

Costs for Resources Used			
Resource	Unit	Unit costs (€)	Source
Treatment			
Plaster cast	Procedure	275	NZa & openDIS 2022
Hospital visits			
Emergency department	Visit	258	DCM-2024
Outpatient clinic	Visit	120	DCM-2024
Surgical ward general admission	Visit	580	DCM-2016
Diagnostic Imaging			
Radiography wrist	Procedure	41	Silver Cross Price 2023
Ultrasound wrist	Procedure	87	Silver Cross Price 2023
CT-scan wrist	Procedure	150	Silver Cross Price 2023
MRI-scan wrist	Procedure	265	Silver Cross Price 2023
Electromyography	Procedure	146	Silver Cross Price 2023
Dexascan	Procedure	129	Silver Cross Price 2023
Additional treatment			
Tendon repair	Procedure	2 590	NZa 2022
Carpal tunnel syndrome release	Procedure	685	NZa 2022
Corrective osteotomy	Procedure	2 280	NZa 2022
Medication			
Paracetamol	Tablet	0.17	Pharmacotherapeutic Compass
NSAID	Tablet	0.16	Pharmacotherapeutic Compass
Tramadol	Tablet	0.38	Pharmacotherapeutic Compass
Oxycodon	Tablet	1.53	Pharmacotherapeutic Compass
Primary and personal care			
General practitioner	Visit	31	DCM-2024
Practice nurse	Visit	21	DCM-2024
Physiotherapist	Visit	39	DCM-2024
Company doctor	Visit	181	DCM-2016
Social worker	Visit	127	DCM-2024
Indirect costs			
Productivity loss	Hour	39.88	DCM-2024

*Abbreviations: NZa* Nederlandse Zorgautoriteit, *DIS* dbc-informatiesysteem, *DCM* Dutch Costing Manual for healthcare research

### Statistical analysis

Economic healthcare analyses were performed with an Analyzed-as-Treated protocol. Data on in-hospital care utilities were extracted from the local Electronical Patient Record. Out-of-hospital care expenses, and productivity losses were calculated with patients’ questionnaires. The average costs per patient for available measurements were multiplied by the number of utilities utilized. Total costs per patient were calculated as the sum of all expenses made during the 12-month follow-up period. A complete case analysis was performed initially, with a multiple imputation analysis to address missing data with incomplete follow-up questionnaires. A future hypothetical scenario was analyzed with one ED visit, and only one outpatient clinic room visit instead of two outpatient clinic room visits. This future hypothetical scenario will evaluate the implementation of one week of plaster cast immobilization into the standard-of-care for patients.

Both CEA outcomes costs and QALYs, due to the stepped wedge design, needed to be adjusted for the systematically different observation periods (included as fixed effect in the statistical model), and for clustering in the data fitting an appropriate mixed effects regression (cluster as random effect). Additionally, a set of co-variates were included as fixed effects (age, gender and dominant hand fracture). To account for missing values multiple imputation was applied, assuming missing completely at random. The Cost-Effectiveness plane (CE-plane) and the Cost-Effectiveness Acceptability Curve (CEAC) were inferred from the statistical model described above by using reffects (application in STATA 18) that calculates the best linear unbiased predictions of the random effects (model). Subsequently these predictions of costs and QALY’s were bootstrapped (1000 samples) in a paired way.

The bootstrapping results were visualized in a CE-plane, categorizing differences in costs against differences in QALYs into four quadrants. In the upper right quadrant, the intervention is more costly and more effective, in the upper left quadrant more costly and less effective, in the lower left quadrant cheaper and less effective, and in the lower right quadrant cheaper and more effective. A CEAC was created to visualize the uncertainty surrounding the cost-effectiveness of one week of cast immobilization at different values of willingness to pay.

Descriptive analysis for the outcomes are presented with statistical parameters, including Standard Deviation (SD), Confidence Interval (CI), P-values, and analyzed with an independent T-test. To address potential confounding factors, a linear mixed model was used with a random effect for cluster and fixed effects for periods, considering the cluster effect introduced by hospitals. The analysis was adjusted for secular trends regarding the stepped wedge design, age, gender, and presence of fractured dominant hand.

The original stepped wedge design in this study included 11 hospitals, 10 clusters and 12 periods (Appendix B). A sample size of 330 patients was calculated with a power of 0.85, alfa of 0.05 and intra-cluster correlation (ICC) of 0.01. The results from the Cast-OFF trial were used for the reference values [5]. For the Cast-OFF 2 study, the primary outcome was the Patient Rated Wrist Evaluation score after six weeks. The PRWE measures wrist pain and disability in daily activities using several subscales ranging from 0–10 points with a total overall score of 100. Higher overall outcomes represents worse functional outcomes [25]. A PRWE score of 35 for the control group and 25 for the intervention group was used for the sample size calculation. A difference of around 11 points is needed for a relevant clinical difference [26]. To account for 30% lost to follow-up, as seen in the Cast-OFF trial, a sample size of 440 patients was calculated., i.e. 4 patients per cluster per month.

Statistical analysis was performed with IBM SPSS, Version 27.0.1.0, and STATA version 18.

## Results

**Fig. 1:**
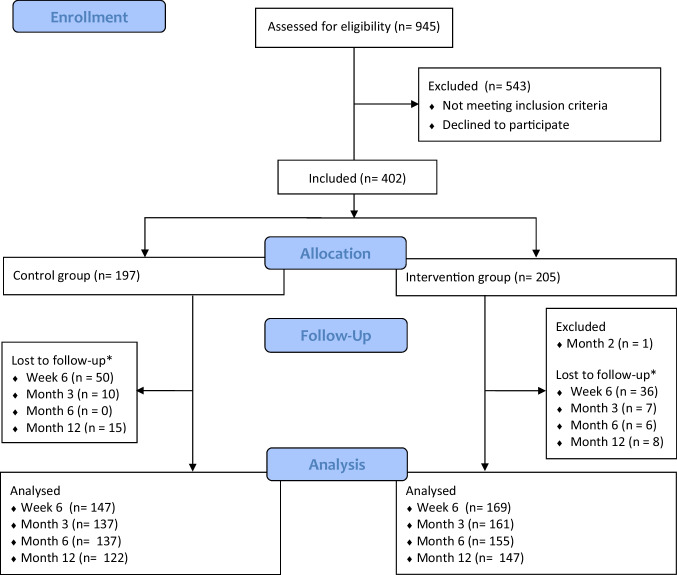
CONSORT FLOW diagram. Number of patients screened, included, lost to follow-up and analyzed. *Lost of follow-up due to withdrawal from study or no reasons given

Between January 2022 and January 2023, 402 patients were included in 11 Dutch hospitals, from which 197 patients in three to five weeks of immobilization group and 205 patients in one week immobilization group. Lost to follow-up occurred in both groups at week six, month three, six, and twelve (Fig. 1). Crossover took place in ten patients, six patients towards the control group, and four patients towards the intervention group. One patient was excluded after two months due to a new trauma of the same wrist. There were no statistically significant differences among patient baseline characteristics (Table 3). In total, 256 patients (64%) completed all questionnaires for a complete case scenario.

**Table 3:** Patient baseline characteristics and complications

	Control group (*n = *197)	Intervention group (*n = *205)	*P*-value
Baseline characteristics			
Age, y (SD)	53.7 ± 18.6	53.3 ± 19.5	0.27
Female	129 (66%)	138 (67%)	0.44
Dominant hand fractured	87 (44%)	109 (53%)	0.39
Extra-articular fracture	77 (39%)	84 (41%)	0.44
Complications			
Operation • Corrective osteotomy • Tendon rupture surgery • CTSR	3 (1,5%) 1 (0,5%) 1 (0,5%) 1 (0,5%)	3 (1,5%) 0 2 (1,0%) 1 (0,5%)	0.91

Abbreviations: *y* year, *SD* Standard Deviation, *CTSR* carpal tunnel syndrome release

### Scenario analysis

Multiple scenarios were analyzed. One from a healthcare perspective with both a complete case scenario, and a multiple imputation scenario. Furthermore, a societal perspective was analyzed with healthcare costs, and loss of productivity costs, both for a complete case scenario, and multiple imputation scenario. Additionally, for both scenarios, a future hypothetical perspective was assessed with reduction of one outpatient clinic visit.

#### Healthcare perspective

The complete case scenario involving 256 patients showed a QALY of + 0.02 for the intervention group (control 0.80 vs 0.82 intervention, [CI −0.03, 0.07]). Savings were made for the intervention group, although not statistically significant. Healthcare savings amounted to €148.76 for the complete case scenario, and €221.25 for the multiple imputation scenario. Significant healthcare savings were observed in the future hypothetical perspective scenario with €254.27 [CI −467.27, −41.21, P 0.02] (Table 4).

**Table 4:** Healthcare perspective in a complete case, and multiple imputation scenario. Both scenarios were presented with the current standard-of-care and the future hypothetical perspective. P-values are given for the differences in QALY and costs

	Difference	Control	Intervention	P
Complete case (*n = *256)				
QALY [CI]	0.02 [−0.03, 0.07]	0.80 [0.77, 0.84]	0.82 [0.80, 0.85]	0.42
Current clinical setting [CI]	€148.76 [−362.07, 64.54]	€1431.80 [1248.57, 1615.03]	€1283.03 [1147.67, 1418.39]	0.17
Future hypothethical perspective [CI]	€254.27 [−467.33, −41.21]	€1436,47 [1251.55, 1621.39]	€1182.20 [1045.02, 1319.39]	0.02
Multiple imputation (*n = *402)				
QALY [CI]	0.021 [−0.02, 0.07]	0.81 [0.78, 0.84]	0.83 [0.81, 0.85]	0.36
Current clinical setting [CI]	€221.25 [−587.02, 144.52]	€1447.60 [1191.73, 1703.45]	€1226.34 [1030.12, 1422.57]	0.22
Future hypothethical perspective [CI]	€322.41 [−687.48, 42.65]	€1449.38 [1193.60, 1705.17]	€1126.97 [930.78, 1323.16]	0.08

*Abbreviations: QALY* Quality-Adjusted Life Years, *CI* confidence interval

#### Societal perspective

The complete case scenario showed a QALY of + 0.02 for the intervention group (control 0.80 vs 0.82 intervention, [CI −0.03, 0.07]). Cost savings were observed, amounting to €31.94 for the complete case scenario, and €182.48 for the multiple imputation scenario. Future hypothetical perspective analysis showed total savings of €137.37, and €283.62 for respectively complete case, and multiple imputation scenario (Table 5).

**Table 5:** Societal perspective for the complete case, and multiple imputation scenario. Both scenarios were presented with the current standard-of-care and the future hypothetical perspective. P-values are given for the differences in QALY and costs

	Difference	Control	Intervention	P
Complete case (*n = *256)				
QALY [CI]	0.02 [−0.03, 0.07]	0.80 [0.77, 0.84]	0.82 [0.80, 0.85]	0.42
Current clinical setting [CI]	€31.94 [−937.97, 874.01]	€2413.43 [1662.30, 3164.56]	€2381.48 [2014.87, 2748.10]	0.95
Future hypothethical perspective [CI]	€137.37 [−1049.56, 774.83]	€2417.63 [1163.36, 3171.91]	€2280.27 [1911.19, 2649.35]	0.77
Multiple imputation (*n = *402)				
QALY [CI]	0.02 [−0.02, 0.06]	0.81 [0.79, 0.84]	0.83 [0.81, 0.85]	0.36
Current clinical setting [CI]	€182.48 [−1371.19, 1006.23]	€2515.80 [1730.70, 3300.90]	€2333.33 [1719.86, 2946.79]	0.76
Future hypothethical perspective [CI]	€283.62 [−1473.63, 906.39]	€2517.55 [1731.92, 3303.18]	€2233.93 [1619.75, 2848.11]	0.63

*Abbreviations: QALY* Quality-Adjusted Life Years, *CI* confidence interval

#### Cost-effectiveness plane

**Fig. 2:**
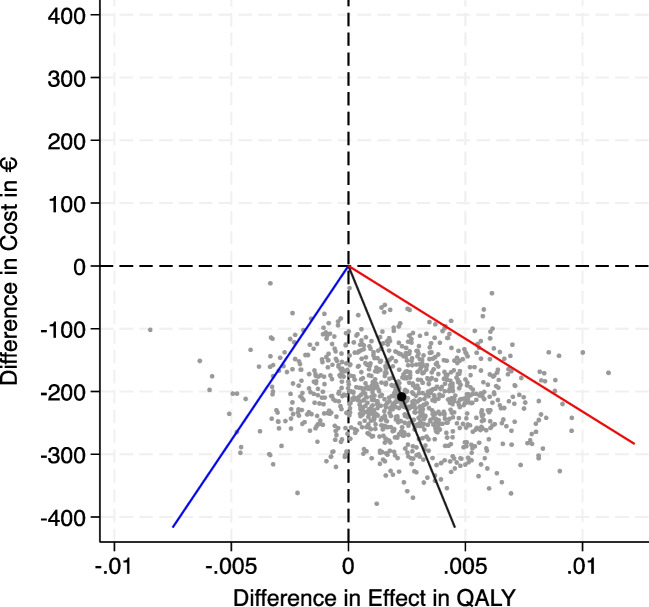
Cost-effectiveness plane. Cost-effectiveness plane for the societal perspective scenario showing differences in societal costs, and effect of intervention in quality adjusted life years (QALY). In the upper right quadrant, the intervention is more expensive and more effective, in the upper left quadrant more expensive and less effective, in the lower left quadrant the intervention is less expensive and less effective, in the lower right quadrant less expensive and more effective. Values between the red and blue line indicate the 95% confidence interval

The cost-effectiveness plane illustrates the variances in societal costs along the y-axis, and effect of the intervention along the x-axis (QALY), based on 1000 bootstrap samples. The figure indicates that the intervention demonstrated cost-effectiveness in the majority of the samples, with the bootstrapped mean in the right lower quadrant. All simulations are beneath the x-axis meaning cost saving for all samples (Fig. 2).

#### Cost-effectiveness acceptability curve

**Fig. 3:**
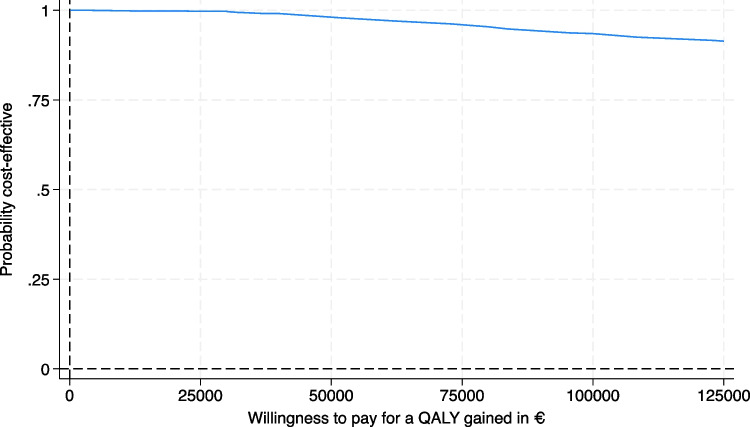
Cost-effectiveness acceptability curve. Cost-effectiveness acceptability curve showing the probability of one week of cast immobilization being cost-effective for different values of willingness to pay for a QALY gained

The probability that the intervention is cost-effective compared to standard care is high for a wide range of societal willingness to pay threshold, up to €125.000,- for a QALY gained (Fig. 3). An increased willingness to pay means the more society is willing to pay for the intervention with one week of cast immobilization.

## Discussion

This CEA was part of the Cast-OFF 2 study where one week of plaster cast immobilization for non-reduced DRF is investigated Savings for the intervention group ranged from €31.94 to €322.41 depending on different scenarios. To date, little research has been performed regarding cost-effectiveness with different durations of plaster cast immobilization for the non- or minimally displaced DRF, making this study difficult to compare to existing literature. Most cost effectiveness studies compared nonsurgical with surgical treatment of patients with DRFs. From these studies, most authors concluded that nonsurgical treatment was more cost-effective in comparison to surgical treatment [27, 28]. Franovic et al. (2023) conducted a large study comparing the cost-effectiveness of nonsurgical management, external fixation, percutaneous pinning, and plate fixation for DRFs. Nonsurgical treatment was consistently associated with lower costs compared to the other treatment options [13].

The clinical results of the Cast-OFF 2 study revealed comparable outcomes in pain, complications, and additional surgical treatment between one week and three to five weeks of cast immobilization. Moreover, functional outcomes were either equivalent or superior. This suggests the potential feasibility of implementing one week of cast immobilization in national guidelines or discussing this treatment option with eligible patients. Adopting one week of cast immobilization for the non- or minimally displaced DRF could lead to reduced hospital visits, with only two required: one initial ED visit, and one outpatient clinic room visit after one week where cast removal is performed. The analyzed complete case healthcare scenario showed significant cost savings with only two hospitals visits. Notably, in the Cast-OFF 2 study, patients from both groups returned for further evaluation if persistent impaired function or pain is observed after the last follow-up appointment. Furthermore, in the intervention group, some patients already did not attend their scheduled second follow-up appointment after cast removal. Some patients in the intervention group already found it burdensome to attend the second follow-up appointment for a physical evaluation as they experienced no pain or limitation in function.

This study has several strengths. Firstly, it is the largest cost-effectiveness analysis investigating one week of plaster cast immobilization for the non- or minimally displaced DRF. Previous studies primarily focused mainly on the conservative treatment compared to surgical treatment. This study included 11 participating hospitals, both academic, and peripheral, reflecting the diversity of the patient population in the Netherlands. This diversity enhances the generalizability of the results, making them applicable for implementation in the Dutch guidelines, and healthcare system. Another additional strength can be found in the future perspective scenario in this study, involving a reduction in outpatient hospital visits for the intervention group. Although not yet implemented, one ED visit and one outpatient clinic visit instead of two outpatient clinic visits, can significantly reduce healthcare expenses. A reduction of one outpatient clinic visit can yield substantial cost savings per patient with a minimum of €120, while also reducing the burden on healthcare personnel, and minimizing patient-associated expenses such as travel costs, time off work, or arrange childcare. While this €120 reduction is a key cost driver, total savings may exceed this amount, due to reductions in diagnostics, follow-up procedures, productivity losses, and patient-associated expenses. During this study, some patients in the intervention group were unsatisfied with the necessity of the second outpatient clinic hospital visits, particularly when being asymptomatic, leading to non-compliance with scheduled appointments. This highlights the importance to tailor healthcare to patient needs and preferences.

This study had several limitations. Firstly, costs for treatment can differ among different hospitals, and countries. Although efforts were made to utilize mean costs for both academic, and peripheral hospitals in the Netherlands, precise estimations for additional surgical interventions were challenging due to the lack of clear pricing data. Costs for surgical interventions were derived from a Dutch database, although local hospitals may have used different costs [18, 19]. Secondly, differences in local hospital organization, such as direct presentation to the outpatient clinic rooms compared to ED visits, could introduce bias in cost assessment. All initial hospital visits were categorized as ED visits in this study, potentially overestimating costs for patients visiting outpatient clinic rooms during regular hours. Thirdly, complete case scenario was not available for all patients. Some patients found the follow-up questionnaires burdensome, or did not have any complaints, leading to withdrawal from follow-up. The use of multiple imputation to address for these missing data may have caused potential bias. Furthermore, some patients misinterpreted the questionnaires, and filled these out with daily complaints unrelated to their injured wrist. Therefore potentially overestimating limitations due to the injured wrist. A 12 month follow-up period was used for this CEA. This duration of follow-up is widely accepted for fracture care, but may not capture all relevant expenses, especially for patients with persistent symptoms after 12 months. Additionally, costs for medication was calculated based on prescribed medication. Medication such as Paracetamol, and Ibuprofen are commonly bought as over-the-counter medication, and are cheaper compared to prescribed medication. Indexing of costs to index year 2022 may have resulted in an overestimation, as not all treatment costs may have been affected by inflation.

Despite the limitations, one week of plaster cast immobilization has the potential to contribute to lower healthcare expenses in the Netherlands. Given comparable or better cost savings, and functional outcome, treating physicians can consider adopting one week of cast immobilization as new standard-of-care.

## Conclusion

This study demonstrated the potential of cost savings with one week of plaster cast immobilization for non- or minimally displaced DRF compared to standard care. Additionally, with comparable effects in QALYs, adopting one week of cast immobilization as the new standard-of-care has the potential to reduce healthcare costs.

## Data Availability

Data will be made available upon reasonable request.

## Appendix A

Participating hospitals: Radboud university medical center, Maastricht university medical center, Canisius Wilhemina hospital, Medical Spectrum Twente, Meander Medical center, Catharina hospital, Maasziekenhuis Pantein, Gelderse Vallei hospital, Slingeland hospital, VieCuri hospital and Rijnstate hospital.

## Appendix B

See Table 6.

**Table 6:** Cast-OFF 2 original planned stepped wedge design. Protocol A: control group, three to five weeks of cast immobilization. Protocol B: intervention group, one week of cast immobilization. Cluster 1–10 represents 11 hospitals

Cluster	T1	T2	T3	T4	T5	T6	T7	T8	T9	T10	T11	T12
1	A	A	B	B	B	B	B	B	B	B	B	B
2	A	A	A	B	B	B	B	B	B	B	B	B
3	A	A	A	A	B	B	B	B	B	B	B	B
4	A	A	A	A	A	B	B	B	B	B	B	B
5	A	A	A	A	A	A	B	B	B	B	B	B
6	A	A	A	A	A	A	B	B	B	B	B	B
7	A	A	A	A	A	A	A	B	B	B	B	B
8	A	A	A	A	A	A	A	A	B	B	B	B
9	A	A	A	A	A	A	A	A	A	B	B	B
10	A	A	A	A	A	A	A	A	A	A	B	B

Abbreviaons: *T* Time in months. See Appendix B for adjusted stepped wedge design

## Declarations

### JEL code

I20

### Note

The study was performed at the Radboud University Medical Center, Nijmegen, the Netherlands.
